# Complete Genome Sequence of *Caulobacter* sp. Strain S6, Obtained Using Oxford Nanopore and Illumina Sequencing

**DOI:** 10.1128/MRA.00680-21

**Published:** 2021-09-30

**Authors:** Cuiyang Zhang, Jun Huang, Wei Ouyang, Yongmei Li, Ying Tang, Zhaohui Guo, Ping Lei, Qingshu Liu

**Affiliations:** a Hunan Institute of Microbiology, Changsha, Hunan, China; SIPBS, University of Strathclyde

## Abstract

We report the complete genome sequence of *Caulobacter* sp. strain S6, generated from Oxford Nanopore and Illumina sequencing. The assembled genome comprises a chromosome with a length of 5.5 Mb and a plasmid of 96,014 bp.

## ANNOUNCEMENT

*Caulobacter* sp. strain S6 was isolated from a soil sample collected on a rocky mountain in Changsha, Hunan Province, China (28.46°N, 113.18°E). The soil sample was diluted and spread onto DSMZ medium 1426, using tryptone instead of peptone (1). After 6 days, one pale colony designated S6 was chosen for further purification. The isolate was subcultivated routinely on the modified DSMZ medium 1426 at 28°C.

The 16S rRNA gene of S6 was obtained by PCR amplification using the primers 27F and 1492R (2) and then ligated to the pMD-18T vector via TA Cloning for sequencing. The sequence was subsequently analyzed using the EzBioCloud server, revealing its closest relatedness to Caulobacter henricii ATCC 15253 with a similarity of 96.15%, which is below the threshold of 98.7% for differentiating two species (3, 4). Currently, the genus *Caulobacter* contains 12 species with validly published names (5–7). The whole-genome sequencing of S6 is essential for fully understanding the taxonomy and novel biosynthetic pathways.

After purity confirmation, a fresh culture of S6 was collected and rinsed for genomic DNA extraction using a standard phenol-chloroform method. The resulting extract was further purified using AMPure XP beads (Beckman Coulter) and then quantified and quality controlled using a Qubit 2.0 fluorometer (Thermo Fisher Scientific), NanoDrop software, and agarose gel electrophoresis (8). For Oxford Nanopore sequencing, high-molecular-weight DNA was isolated using the BluePippin system (Sage Science, Beverly, MA, USA). The Oxford Nanopore Technologies (ONT) library was constructed from approximately 1.5 μg genomic DNA using the one-dimensional (1D) ligation sequencing kit (SQK-LSK109; ONT). No size selection or shearing was applied. The library was loaded onto a R9.4 flow cell for the PromethION platform (PromethION flow cell, FLO-PRO002; Oxford Nanopore). Albacore v4.3.2 was used for base calling, yielding a total of 25,093 reads with an average length of 39,853 bp and an *N*_50_ value of 41,325 bp (9). Nanopore quality control was achieved using NanoPlot v1.15.0 with a threshold quality (Q) value of >7 (10).

For Illumina sequencing, around 1 μg genomic DNA, prepared as described above, was used with the NEBNext Ultra DNA library prep kit (New England Biolabs), according to the manufacturer’s instructions. The Illumina library was sequenced on the Illumina NovaSeq 6000 platform at Benagen (Wuhan, China). Approximately 1.7 Gb of raw data, comprising 150-bp long paired-end reads, were generated. A total of 6,552,806 reads were subjected to quality control and trimming using SOAPnuke v1.3.0 (11), which removed reads containing 50% low-quality bases (quality value, ≤5) and overlaps with adapter sequences, generating 6,545,540 clean reads.

The assembly was completed using Unicycler v0.4.8 (12), yielding 2 circular contigs with lengths of 5,553,470 bp and 96,014 bp and GC contents of 67.81% and 68.28%, respectively. The final coverage of the genome is 100%. The depth of Illumina sequencing was on average 346.26×, while for Nanopore it was 172.73×. For all software used, default parameters were used, except where otherwise noted. Annotation was performed using the NCBI Prokaryotic Genome Annotation Pipeline (PGAP) v4.12 (13), which predicted a total of 5,151 genes with 5,079 protein coding sequences, 57 RNA genes, and 15 pseudogenes.

### Data availability.

The complete genome sequence of *Caulobacter* sp. strain S6 has been deposited at GenBank under accession numbers CP073078 and CP073079. The raw reads have been deposited at the SRA under accession numbers SRR14173250 and SRR14173251. The BioSample and BioProject accession numbers are SAMN18580201 and PRJNA718988, respectively.
